# Cigarette Smoking and Brain Regulation of Energy Homeostasis

**DOI:** 10.3389/fphar.2012.00147

**Published:** 2012-07-25

**Authors:** Hui Chen, Sonia Saad, Shaun L. Sandow, Paul P. Bertrand

**Affiliations:** ^1^Faculty of Science, School of Medical and Molecular Biosciences, University of TechnologySydney, NSW, Australia; ^2^Faculty of Medicine, Department of Pharmacology, School of Medical Sciences, University of New South WalesSydney, NSW, Australia; ^3^Renal Research Group, Kolling Institute, University of SydneySydney, NSW, Australia; ^4^Faculty of Medicine, Department of Physiology, School of Medical Sciences, University of New South WalesSydney, NSW, Australia

**Keywords:** smoking, nicotine, appetite regulation, reward, programming

## Abstract

Cigarette smoking is an addictive behavior, and is the primary cause of cardiovascular and pulmonary disease, and cancer (among other diseases). Cigarette smoke contains thousands of components that may affect caloric intake and energy expenditure, although nicotine is the major addictive substance present, and has the best described actions. Nicotine exposure from cigarette smoke can change brain feeding regulation to reduce appetite via both energy homeostatic and reward mechanisms, causing a negative energy state which is characterized by reduced energy intake and increased energy expenditure that are linked to low body weight. These findings have led to the public perception that smoking is associated with weight loss. However, its effects at reducing abdominal fat mass (a predisposing factor for glucose intolerance and insulin resistance) are marginal, and its promotion of lean body mass loss in animal studies suggests a limited potential for treatment in obesity. Smoking during pregnancy puts pressure on the mother’s metabolic system and is a significant contributor to adverse pregnancy outcomes. Smoking is a predictor of future risk for respiratory dysfunction, social behavioral problems, cardiovascular disease, obesity, and type-2 diabetes. Catch-up growth is normally observed in children exposed to intrauterine smoke, which has been linked to subsequent childhood obesity. Nicotine can have a profound impact on the developing fetal brain, via its ability to rapidly and fully pass the placenta. In animal studies this has been linked with abnormal hypothalamic gene expression of appetite regulators such as downregulation of NPY and POMC in the arcuate nucleus of the hypothalamus. Maternal smoking or nicotine replacement leads to unhealthy eating habits (such as junk food addiction) and other behavioral disorders in the offspring.

## Introduction

Cigarette smoking is the leading preventable cause of death and disability from respiratory disease. Smoking causes addiction and is negatively correlated with body weight and caloric intake; an effect which appears to be nicotine-mediated (Hajek et al., 1988). It is this action of nicotine on energy homeostasis that is attracting attention as a potential weight loss treatment during the current global obesity pandemic. However, the fat loss associated with nicotine has not been confirmed in human subjects under well-controlled experimental conditions. This review will decipher the neurophysiological mechanisms that underlie the regulation of cigarette smoking/nicotine on energy homeostasis based on both animal and human studies. The impact of maternal smoking on fetal energy homeostatic regulation will also been discussed, as there is a relatively high rate of smoking during pregnancy. Finally, whether or not nicotine is a good candidate as a weight loss treatment will be discussed.

## Cigarette Smoking and Weight Control

Cigarette smoking is an addictive behavior with the consequences being the leading preventable cause of death and disability worldwide. It is a primary cause of cancer and cardiovascular and pulmonary disease. There are >1 billion people who smoke around the world (DeMarini, 2004), with ∼6 million deaths each year being due to tobacco/cigarette smoking-related disease; resulting in significant social and economic cost to Society (World Health Organization, 2011). It has been estimated that in less than 40 years, deaths due to smoking-related illness will rise to ∼10 million per year (DeMarini, 2004; Hussein et al., 2007).

Smoking induces a negative energy state, characterized by reduced energy intake and body weight, which has been well documented across species (Perkins, 1992; Strauss and Mir, 2001; Bellinger et al., 2003; Fulkerson and French, 2003; Chen et al., 2006, 2007, 2008). The lowered body weight has been shown to be independent of diet type, with a similar proportion of weight loss displayed in mice consuming a diet with either low or high-fat concentrations after 7 weeks of cigarette smoke exposure (Chen et al., 2007). Unfortunately, these and similar observations have led to the public perception that smoking is associated with weight loss, and it is commonly used as a weight control strategy, especially among the young, and females (Camp et al., 1993; Wiseman, 1998; Fulkerson and French, 2003). Weight gain and increased craving for high caloric junk food on cessation of smoking without nicotine supplementation is one of the reasons given by people that prevents them from ceasing smoking (Stamford et al., 1986; Grunberg et al., 1988; Filozof et al., 2004), and this is also supported by the literature, with >75% of former smokers gaining weight after cessation (Williamson et al., 1991; Leischow et al., 1992).

Cigarette smoke contains at least 6000 components that may directly or indirectly affect caloric intake and energy expenditure. Nicotine, the major addictive substance within cigarette smoke, is the best described for its suppressive effects on body weight and appetite in both humans and animal models (Wager-Srdar et al., 1984; Grunberg et al., 1986; Bellinger et al., 2003). Furthermore, cigarette smoke stimulates the inflammatory response associated with elevated circulating levels of inflammatory cytokines, such as tumor necrosis factor α and interleukin 6, which are associated with the development of disease states related to smoking (Fernandez-Real et al., 2003). These cytokines have been shown to inhibit appetite and affect lipid metabolism (Langhans and Hrupka, 1999; Jansson et al., 2003). Overall, studies using cigarette smoke exposure have improved insight into the effects of cigarette smoking-related anorexia and weight loss.

An important question that arises from such studies is whether lower caloric intake is the main contributor to the generally lower body weight in smokers. This question can be answered by the use of pair-fed animals, which receive the same amount of food as that consumed by smoke-exposed litter-mates. According to the results of such studies, the weight loss effects of cigarette smoke exposure were not only due to the predicted reduction in energy intake, but also to an enhanced capacity for energy expenditure (Chen et al., 2006, 2008). Increased energy expenditure and thermogenesis can occur when the proton gradient of the inner mitochondrial membrane dissipates; a state which occurs via the action of mitochondrial carrier proteins termed uncoupling proteins (UCPs; Dalgaard and Pedersen, 2001). Uncoupling of the mitochondrial proton gradient is thought to be important for the maintenance of cellular respiration, activation of substrate oxidation, and prevention of the generation of reactive oxygen species (Lee et al., 1999). There are several homologs of UCPs including UCP1, which, when active in brown fat is responsible for non-shivering thermogenesis in newborn humans, in cold acclimatization, and hibernating mammals (Cannon and Nedergaard, 2004). In contrast, UCP3 is implicated in the regulation of shivering and other forms of thermogenesis, mitochondrial fatty acid transport, and basal metabolic rate (Samec et al., 1998; Argyropoulos and Harper, 2002; Schrauwen and Hesselink, 2003). Fasting or chronic food restriction normally results in the downregulation of UCP1 expression in brown fat (Champigny and Ricquier, 1990) while nicotine induces UCP1 mRNA expression, which likely leads to enhanced energy expenditure (Yoshida et al., 1999; Arai et al., 2001). In mice directly exposed to cigarette smoke, both UCP1 and three mRNA expression was increased compared with pair-fed animals (Chen et al., 2006, 2008), suggesting that increased energy expenditure occurred despite their reduced energy intake. This theory has also been supported by data from humans, where energy expenditure was increased by nicotine administration (Perkins et al., 1989).

## Cigarette Smoking and Adiposity

Although smokers are generally thought to weigh less than non-smokers, smoking is actually a predisposing factor for abdominal obesity, glucose intolerance, and insulin resistance (Canoy et al., 2005; Chen et al., 2007), which is a situation not well recognized by the general public. In a rodent model, we have shown that the reduction in fat mass after cigarette smoke exposure occurred only if the mice consumed a low-fat balanced diet. In addition, this weight loss was accompanied by lean body mass wasting, including that associated with some major organs such as liver, kidney, and skeletal muscle (Chen et al., 2005, 2006, 2008). Cigarette smoke exposure failed to cause fat loss when the mice consumed a high-fat cafeteria style diet consisting of foods such as fried potatoes, cakes, and sweet biscuits; whereas lean body mass loss became the prominent cause of weight loss in these mice (Chen et al., 2007). We speculate that this observation was due to a change of food preference induced by cigarette smoke exposure or, perhaps that the nature of the high-fat diet to induce over accumulation of fat mass, even with restricted caloric intake. In both human and animal studies, food high in refined sugar and fat is more preferred when they are exposed to cigarette smoke (Marangon et al., 1998; Chen et al., 2007). Consuming such food can increase fat mass, blood lipid levels, and glucose intolerance even when the total calorie intake does not exceed the daily requirement (Shiraev et al., 2009). In contrast, when smoke-exposed mice consume a high-fat diet, they consume twice the energy of the recommended daily requirement (Chen et al., 2007). Thus, we can speculate that adiposity induced by consumption of a high-fat diet, together with the loss of lean body mass found exclusively after cigarette smoke exposure may increase the risk of metabolic disorders.

In fact, both active and passive smoking contribute to glucose intolerance and insulin resistance, leading to type-2 diabetes; and smoking cessation has been demonstrated to improve insulin sensitivity (Facchini et al., 1992; Eliasson et al., 1997). It has been suggested that insulin resistance among smokers may be due to the direct impact of nicotine, carbon monoxide, or other agents in the tobacco smoke (Facchini et al., 1992). Nicotine infusion stimulates lipolysis to increase triglyceride levels in both human and animal studies (Sztalryd et al., 1996; Andersson and Arner, 2001), while hyperlipidemia is strongly associated with the onset of insulin resistance (Stannard and Johnson, 2004). Anorexia developed in long-term smokers also contributes to muscle wasting, especially in those with chronic obstructive pulmonary disease (Morrison et al., 1988; Jagoe and Engelen, 2003). Skeletal muscle is one of the major sites for insulin-dependent glucose deposition when blood glucose rises. Thus, in smokers, the reduction in muscle mass can directly impair systemic glucose uptake, contributing to postprandial hyperglycemia, and an elevated risk of developing type-2 diabetes. Vascular changes associated with prolonged smoking may also lead to reduced blood flow to skeletal muscle and decreased insulin-mediated glucose uptake (Facchini et al., 1992).

## Neurological Mechanisms Underlying Suppressed Appetite

### Classical feeding regulators

The reduction in energy intake associated with smoking shows a relationship to the effects of several brain appetite regulators, and indeed, nicotinic receptors have been demonstrated in the appetite regulating area of the hypothalamus (Jo et al., 2002). The most widely studied appetite regulator is neuropeptide Y (NPY), a 36 amino acid peptide. NPY is a member of the pancreatic polypeptide family, and is abundant throughout the central nervous system and the periphery (Tatemoto et al., 1982; Allen et al., 1983). NPY is a powerful neurochemical stimulator of feeding in many species (Vettor et al., 1994; Raposinho et al., 2001), with its levels reflecting the nutritional status of the body, and contributing to the long-term regulation of energy homeostasis. Administration of NPY into different brain regions, including the hypothalamus, frontal cortex, hindbrain, and hippocampus, induces hyperphagia (even in a satiated state), decreased sympathetic activity and thermogenesis, increased fat deposition, and promotion of weight gain and obesity (Clark et al., 1984; Billington et al., 1991; Egawa et al., 1991; Raposinho et al., 2001).

In studies of a mouse model of cigarette smoke exposure, the hypothalamic NPY concentration was significantly suppressed by smoke exposure, compared with food restriction (pair-feeding; Chen et al., 2006, 2008). This effect appears to be predominately nicotine-mediated, as a similar suppression of NPY has been observed in nicotine-treated animals (Jo et al., 2002). Physiologically, the decreased hypothalamic NPY levels can upregulate the expression of orexigenic NPY receptors. However, the hypothalamic density of the NPY Y_1_ receptor is reduced by chronic nicotine treatment (Kane et al., 2001). Thus, it is possible that a voluntary reduction in energy intake in smokers can be attributed to suppressed NPY signaling in both the presynaptic production of the peptide and at the postsynaptic receptor level. This inhibitory effect of nicotine on appetite may be an important clue for therapy development for the treatment of obesity. This is of significant relevance, as clinical trials targeting NPY pathways have failed in obese patients due to redundancy in the mechanisms regulating energy homeostasis.

Neuropeptide Y is not the only neuropeptide in the central nerve system that can regulate appetite and energy balance. Agouti-related protein (AgRP) is another potent orexigenic molecule, which co-localizes with NPY in hypothalamic neurons (Hahn et al., 1998). In addition, there are also melanocortins, including adrenocorticotropin and melanocyte-stimulating hormones (MSH), which are peptide cleavage products of proopiomelanocortin (POMC) and exert their effects by binding to the melanocortin receptors (MCRs). The melanocortin system is thought to be one of the most important pathways involved in food intake and energy regulation, with mutations contributing to ∼4% of genetic obesity in humans (Horvath et al., 2004). Neurons expressing orexigenic NPY and AgRP cooperate with neurons expressing anorexigenic POMC and cocaine-amphetamine-regulated transcript (CART). In the diet-induced obese mouse, when hypothalamic NPY mRNA expression was reduced, AgRP and POMC mRNA were also downregulated (Lin et al., 2000; Wang et al., 2002). This suggests that the anorexigenic neurons containing POMC respond synchronously with orexigenic neurons to maintain the balance between orexigenic and anorexigenic neuropeptides. However, in nicotine-treated mice, the hypothalamic level of CART and POMC derived α-MSH has been shown to be increased (Marty et al., 1985; Kramer et al., 2007), in the face of suppression of NPY and AgRP levels (Chen et al., 2006; Martínez de Morentin et al., 2012). In addition, it has been shown that nicotine withdrawal is linked to increased hypothalamic NPY and AgRP, although with reduced UCP3 expression (Fornari et al., 2006) resulting in an increased drive to eat, and reduced capacity for energy expenditure.

### Psychological regulators

Feeding is not only controlled by homeostatic mechanisms, which theoretically would allow an individual to maintain an ideal body weight in the long term. Feeding is also controlled by brain reward systems and psychological states, which reinforce the motives for excessive eating without homeostatic value (Saper et al., 2002); namely, those independent of energy expenditure. The consumption of highly palatable foods is now considered to be an addictive behavior (Heilig et al., 1989). In this respect, food and nicotine addiction may share the same central pathways. Addictive eating behavior has been suggested to be predominantly controlled by the interactions between the classical “feeding center” in the lateral hypothalamus and the nucleus accumbens within the mesolimbic system, and coordination between the neurotransmitters, such as dopamine, serotonin, and the opioid system (Saper et al., 2002). Nicotine administration releases dopamine in many brain regions involved in reward, such as the mesolimbic area, the corpus striatum, the frontal cortex, and ventral tegmental area in the brain stem (Gilbert et al., 1989; Benowitz, 2010). Increased brain release of serotonin and endogenous opioid peptides, as well as the upregulation of opioid receptors, have also been reported in various animals models following nicotine administration (Marty et al., 1985; Martínez de Morentin et al., 2012). Eating, especially binge eating, is considered to be a physiological reaction to counteract stress in some individuals (Polivy et al., 1994). Nicotine has been shown to reduce anxiety in a dose-dependent manner (Gilbert et al., 1989; Pomerleau and Pomerleau, 2007), which may also overpower the desire to eat, in addition to its suppressive ability of central orexigenic pathways. Nicotine withdrawal can cause anxiety and stress (Picciotto et al., 2002), and both can serve as powerful incentives for former smokers to either overeat or smoke again.

Tolerance due to chronic nicotine use may potentially affect its activation of the brain reward pathway. To date, only the impact of nicotine tolerance on brain dopamine release is well studied, which is also site dependent (Damsma et al., 1989; Izenwasser and Cox, 1992). Nicotine tolerance is only seen in subjective mood effects, such as dizziness and confusion as reviewed by Perkins (2002). However, this tolerance may still lead to an increased demand for nicotine if it is used as an appetite suppressant.

## Smoking during Pregnancy and the Impact on Offspring

**Figure 1:**
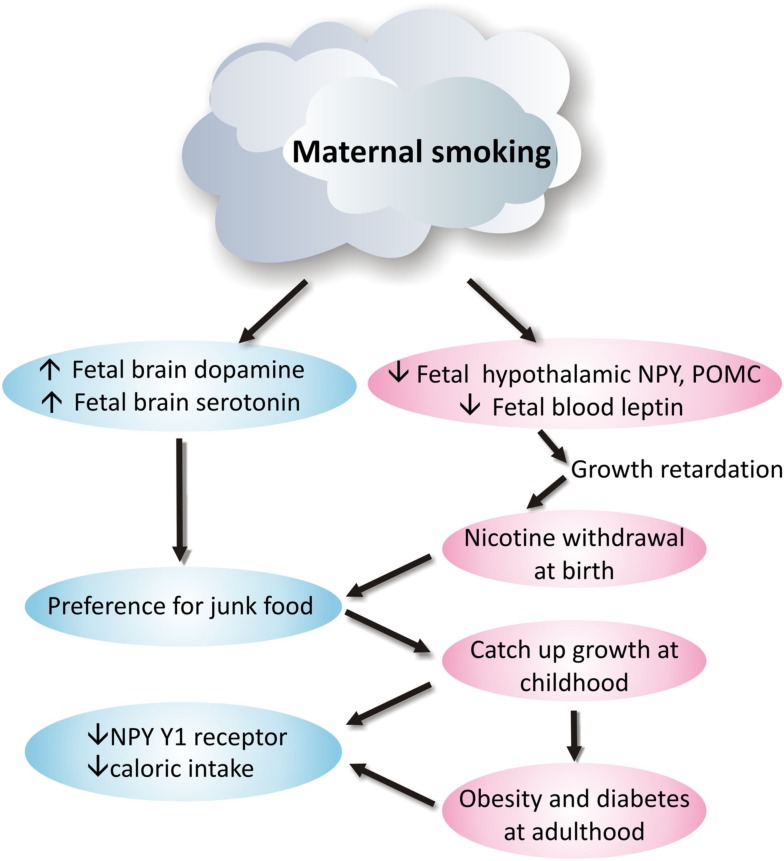
**Neurophysiological mechanism of how maternal smoking programs metabolic disorders in offspring**.

Smoking during pregnancy puts physiological pressure on the mother’s metabolic system and is a significant contributor to adverse pregnancy outcomes, including miscarriage, low birth weight, preterm birth, and perinatal death (Ng et al., 2006; Nielsen et al., 2006; Raatikainen et al., 2007). Moreover, it significantly interrupts fetal development and predicts the future risks for respiratory dysfunction, social behavioral problems, cardiovascular disease, obesity, and type-2 diabetes (Whincup et al., 1989; Orlebeke et al., 1999; Stocks and Dezateux, 2003; Burke et al., 2004; Al Mamun et al., 2006; Bruin et al., 2008b). Despite the disadvantages of maternal smoking, reports still show that ∼25–29% pregnant women smoke during pregnancy (Contal et al., 2005). Some of these processes along with the underlying neurophysiological changes are shown diagrammatically in Figure 1.

### Effects on body weight and eating behavior in offspring

In Western countries, it is maternal smoking during pregnancy rather than poverty that is the major cause of low birth weight (Power and Jefferis, 2002). Even maternal obesity cannot counteract the infant growth retardation due to smoking during pregnancy (Haworth et al., 1980). Studies in humans and other primates suggest that lower birth weight associated with maternal smoking is mainly nicotine-mediated (Haworth et al., 1980; Grove et al., 2001; Collet and Beillard, 2005). However, brain weight does not appear to be affected by intrauterine nicotine exposure (Grove et al., 2001); an observation that may be due to the redistribution of nutrients to preserve brain growth, at the cost of the development of other organs such as the liver and pancreas (Ernst et al., 2001).

Catch-up growth is normally observed in children exposed to intrauterine maternal smoking, and there is evidence linking maternal smoking and childhood obesity in offspring, especially those from the mothers who smoke during early pregnancy (Power and Jefferis, 2002; Al Mamun et al., 2006). It has been reported that children of mothers who smoked during pregnancy started to display an increased risk of being overweight at 5 years of age (Wideroe et al., 2003). Adolescents who are the offspring of mothers who smoked had an increased risk of being among the highest percentile for body mass index (Power and Jefferis, 2002; Al Mamun et al., 2006). Interestingly, smoking cessation after the first trimester does not appear to reduce this risk to the offspring (Toschke et al., 2003), suggesting that the first 3 months of pregnancy are critical for long-term impacts on the wellbeing of the offspring. However, children from former smoking mothers did not show increased risk of obesity (Oken et al., 2005).

Smoking mothers tend to have a shorter breastfeeding period, which deprives the offspring of the protection provided by breast milk against future eating disorders (Gilchrist et al., 2004; Mayer-Davis et al., 2006). On this basis, it can be suggested that the rapid weight gain during the early postnatal period may be due to the effect of nicotine withdrawal, in a similar manner to the increased craving for food and subsequent weight gain seen in smokers after smoking cessation (Lerman et al., 2004). Furthermore, as children also tend to copy the eating habits of their parents, this will be detrimental in the children of smokers, as smokers are more likely to choose foods low in fiber, vitamins and minerals, and high in monounsaturated fatty acids, starch, as well as sugar-sweetened soft drinks (Crawley and While, 1996; Rogers et al., 2003). Indeed, the children of smokers are more likely to be exposed to passive smoking, with ongoing detrimental effects of the chemicals in the cigarette smoke.

### Effects on brain energy homeostatic regulators

Nicotine can have a profound impact on the developing fetal brain, via its ability to rapidly and fully pass across the placenta, with fetal concentrations ∼115% of maternal levels (Walker et al., 1999). When the fetus leaves the womb, the supply of nicotine is removed, and the impact of nicotine withdrawal can be observed in these newborns, as they show increased signs of stress and dysregulation of the hypothalamic-pituitary-adrenal axis (Huizink and Mulder, 2006). Studies in humans, other primates, and mice have observed some neuronal abnormalities relevant to feeding regulation that result from maternal smoking or exposure to nicotine (Mantzoros et al., 1997; Grove et al., 2001; Bruin et al., 2008a). However, the impact of maternal smoking during gestation on brain energy homeostatic pathways in the offspring requires further study.

Maternal smoking is clearly linked to abnormal hypothalamic gene expression of appetite regulators, with NPY and POMC gene expression in the arcuate nucleus of the hypothalamus being significantly downregulated in the newborn primate following intrauterine nicotine exposure (Grove et al., 2001); a state that may reflect an under-developed brain. This state is similar to observations in adult animals with nicotine or cigarette smoke exposure, as clarified above. Indeed, it can be suggested that without the continuing inhibition of nicotine, NPY, and POMC gene expression can rebound to that equal to an early postnatal age, leading to hyperphagia and future obesity. As yet there is no direct data to date to support this hypothesis. However, studies of mouse models have examined the adult offspring from mothers exposed to cigarette smoke and/or those consuming a high-fat diet during the pregnancy (Chen et al., 2011). Surprisingly, despite increased adiposity in offspring from smoke-exposed mothers, their daily caloric intake was actually lower than the offspring from control mothers, regardless of postnatal diet type. Although the levels of POMC were not different between groups, NPY gene expression was only suppressed by maternal consumption of a high-fat diet, and not intrauterine smoke exposure *per se*. However, NPY Y1 receptor gene expression was significantly downregulated by both maternal smoke exposure and a high-fat diet, with this being reflected by reduced food intake in those offspring (Chen et al., 2011). In addition, other components of cigarette smoke, such as carbon monoxide and ingredients in tobacco tar, can also directly affect the fetal brain, and thereby contribute to the above changes in the fetal brain (Ernst et al., 2001). It can be suggested that at adulthood, the changes in brain appetite regulators may be an adaptation to increased adiposity, rather than a prolonged impact of intrauterine smoke exposure.

Another important appetite regulator is the adipocyte-derived hormone leptin, which is critical for the development of neurons and neural projections between hypothalamic nuclei involved in appetite control in early life (Bouret et al., 2004). In mice, a lack of leptin during the early postnatal period results in sparse neuronal projections in the hypothalamus, and later in life, an obese phenotype (Zhang et al., 1994; Chua et al., 1996; Bouret et al., 2004). Leptin supplementation during this early postnatal period can partially restore the reduced hypothalamic neural projections in the leptin-deficient *ob*/*ob* mouse, and partially reverse the hyperphagic phenotype (Bouret et al., 2004). In humans, cord blood leptin concentrations in both full-term and preterm newborns from smoking mothers are reported to be significantly decreased compared to those from non-smoking mothers (Mantzoros et al., 1997). It has been suggested that smoking might increase the production of catecholamines in the infants leading to lipolysis and fat loss, which can be associated with decreased leptin levels (Mantzoros et al., 1997; Ozkan et al., 2005), as circulating leptin levels are in relative proportion to fat mass. In a similar manner, in primates serum leptin levels are reduced by ∼50% in newborns from nicotine-treated mothers compared with those from control mothers (Grove et al., 2001). One hypothesis that may account for this observation is that reduced leptin in newborns from smoking mothers may interrupt the development of the neurons controlling energy homeostasis, contributing to unhealthy eating behavior at adulthood. As with smokers, it may be that the reward pathways override the energy homeostatic control in such offspring, resulting in a preference for junk foods. Studies of offspring from nicotine-treated animals show that dopamine receptor binding affinity is increased, despite reduced receptor density; while brain serotonin turnover was reduced, whilst its transporter was increased in such offspring (Fung and Lau, 1989; Muneoka et al., 1997, 2001). In the original studies of this topic, this finding was used to explain the abnormal social behavioral problems, such as attention deficit hyperactivity disorder or addiction, as found in offspring with intrauterine nicotine exposure. However, changes in the reward pathway may also underlie the unhealthy eating behavior.

## Conclusion

Nicotine can change brain feeding regulation to reduce appetite via both energy homeostatic and reward mechanisms. In animal models, the effects of cigarette smoke exposure on energy homeostasis are clearly both time and dose dependent. As such, the higher the dose, the greater the reduction in caloric intake and body weight. However, the marginal effect of nicotine at reducing abdominal fat in high-fat diet fed animals may shed light on its potential application in the treatment of obesity. Maternal smoking or nicotine replacement can clearly lead to unhealthy eating habits (such as junk food addiction) and other behavioral disorders in the offspring. Thus, smoking cessation without nicotine replacement during pregnancy is recommended. Although the direct use of nicotine for fat loss in the obese is not plausible, the appetite suppressive and energy expenditure promoting effects of nicotine may still be useful. The development of nicotine analogs should be encouraged which avoid addiction, but retain the fat burning-obesity reduction effect.

## Conflict of Interest Statement

The authors declare that the research was conducted in the absence of any commercial or financial relationships that could be construed as a potential conflict of interest.
